# Seasonal Shifts in Primary Water Source Type: A Comparison of Largely Pastoral Communities in Uganda and Tanzania

**DOI:** 10.3390/ijerph13020169

**Published:** 2016-01-27

**Authors:** Amber L. Pearson, Adam Zwickle, Judith Namanya, Amanda Rzotkiewicz, Emiliana Mwita

**Affiliations:** 1 Department of Geography, Michigan State University, East Lansing, MI 48823, USA; namanyaj@msu.edu; 2 Department of Public Health, University of Otago, Wellington 6021, New Zealand; 3 Environmental Science and Policy Program, Michigan State University, MI 48823, USA; zwicklea@msu.edu; 4 School of Criminal Justice, Michigan State University, East Lansing, MI 48823, USA; rzotkiew@msu.edu; 5 Department of Geography and Economics, College of Education, Dar es Salaam University, Dar es Salaam P. O. Box 2329, Tanzania; emmyrh@yahoo.com

**Keywords:** access, seasonal, water source, pastoralists, water quantity

## Abstract

Many water-related illnesses show an increase during the wet season. This is often due to fecal contamination from runoff, yet, it is unknown whether seasonal changes in water availability may also play a role in increased illness via changes in the type of primary water source used by households. Very little is known about the dynamic aspects of access to water and changes in source type across seasons, particularly in semi-arid regions with annual water scarcity. The research questions in this study were: (1) To what degree do households in Uganda (UG) and Tanzania (TZ) change primary water source type between wet and dry seasons?; and (2) How might seasonal changes relate to water quality and health? Using spatial survey data from 92 households each in UG and TZ this study found that, from wet to dry season, 26% (UG) and 9% (TZ) of households switched from a source with higher risk of contamination to a source with lower risk. By comparison, only 20% (UG) and 0% (TZ) of households switched from a source with lower risk of contamination to a source with higher risk of contamination. This research suggests that one pathway through which water-related disease prevalence may differ across seasons is the use of water sources with higher risk contamination, and that households with access to sources with lower risks of contamination sometimes choose to use more contaminated sources.

## 1. Introduction

While substantive research on access to water has been conducted, the definition of access to water varies between studies and has evolved within monitoring institutions (e.g., Joint Monitoring Program). In recent decades, much of the water and health research has focused on water quality [1], neglecting other dimensions of water adequacy or access. Typically, measures of access to water include locational access (distance), time (to fetch water, waiting times), financial access (cost of water), microbiological and chemical quality or whether a source is improved/unimproved, availability and reliability (particularly for surface water sources).

Most monitoring of access to water is evaluated cross-sectionally, which limits our understanding of the real, dynamic nature of access. Changes in water sources may vary due to a number of reasons including seasonal variations in rainfall and water availability, maintenance and governance issues which affect accessibility of water, or the eco-social processes which may influence access [2]. Evidence has now emerged that many theoretically year-round water sources are, in reality, often unavailable due to maintenance issues [3] or water inadequacy [4]. For example, among 100 households in Uganda, 30% reported using a year-round water source (e.g., borehole) but were unable to obtain water at least once in the past two weeks [5].

Even with diversity in water access measures, it is clear that inadequate access, however measured, is associated with increased diarrheal, skin and respiratory infections [6]. In fact, in 2012, about 500,000 diarrheal deaths were estimated to be caused by inadequate drinking water [7]. Much of the water and health research in sub-Saharan Africa also indicates a clear increase in infections and symptoms in the wet season, or in times of relative abundance. For example, diarrhea and vomiting in Uganda are prevalent in every season and highest during the wet season [8]. However, this is not true for all infections, as malaria is not markedly seasonal in Uganda [9]. The upward trend in illness in the wet season is, in part, due to water quality issues—increased chemical and/or microbial contamination of water sources from runoff [10]. In fact, numerous outbreaks have occurred and will likely occur into the future related to large rainfall events [11]. In times of scarcity, or the dry season, water inadequacy may negatively influence health [12], whereby lower water usage leads to unhygienic conditions and inadequate consumption, or reliance on sources at greater distances [1] from home or different—and possibly less safe [1]—types of water sources. In arid and semi-arid settings with an extended dry season, it is possible that different water source types may be used in the wet versus the dry season, with implications for health. Yet, very little is known about these aspects of access to water and changes in source type across seasons, particularly in arid regions where water scarcity is an annual event and a major driver of migration and other coping strategies [5].

Recent meta-analyses have also indicated that the likelihood and level of fecal contamination varies by water source type, and by season [13,14,15,16]. Unimproved sources have significantly higher odds of being contaminated compared to improved sources. Even improved sources tend to be contaminated, particularly during the wet season [15]. In the absence of microbiological sampling, these meta-analysis estimates of the proportion of samples ≥ 10 *E. coli* per 100 mL by water source type may be useful proxies of likelihood of contamination as relevant for health. The threshold of 10 *E. coli* per 100 mL is a designation of an “intermediate” water service level as per the World Health Organization (WHO)/ United Nations International Children’s Emergency Fund (UNICEF) working group “ladder” of access [17] and the post-2015 drinking water, sanitation, and hygiene (WASH) targets [18], which may be a more conservative target than ≥ 1 *E. coli* per 100 mL. Given this background, the research questions to be addressed in this study are: (1) To what degree do households in Uganda (UG) and Tanzania (TZ) change primary water source type between wet and dry seasons?; and (2) How might seasonal changes relate to water quality and health? 

## 2. Methods

Two studies on access to water and type of primary household water source used in the wet *versus* the dry season were conducted in Uganda and Tanzania in largely pastoral communities to understand whether source type varies by wet/dry season. In Uganda, in-person surveys were carried out in three communities located in the south-western semi-arid savannah in Kiruhura District in 2009 during the start of the dry season in May. Households were selected in stages. First three villages were randomly selected from village clusters in Kiruhura District. Then, households were randomly selected from using proportional population sampling by village, with a goal of 100 households total. Details of the demographics of the sample are provided elsewhere [5].

In Tanzania, similar in-person surveys were conducted in one Maasai community in the Arusha region, Monduli District, in 2015 during the start of the dry season in July. Households were randomly selected from one village, using Google Earth to delineate the sample frame, as outlined in detail elsewhere [19]. Both regions have an annual extended dry season and few improved water sources. The surveys included identical questions about access to household water sources across seasons.

Specifically, households were asked: “In the wet season, what is your primary water source for your household purposes?” and “In the dry season, what is your primary water source for your household purposes?” Distinctions were made between household and livestock or agricultural purposes. After the name of the source was given, the type of source was asked and verified in the field, by visiting each water source (or location if evaporated) identified through the surveys. In addition, respondents were asked whether the source is seasonal or year-round (in terms of evaporation) and whether the source was inaccessible for any reason (including maintenance) over the previous two week period. In total, 92 households in Uganda and 92 in Tanzania were surveyed by local enumerators in either Runyankole or Swahili, with either the self-declared head of household or adult present during our visit and asked about water source locations, types and permanence of water availability across seasons. As the survey contained questions beyond water use, we did not strictly ask to speak to the adult in charge of water for the household.

To answer the first research question, counts and proportions of households using the same primary water source type across seasons and those switching source types from the wet to dry season were calculated. We then compared the differences between the proportions of households that switched primary water sources in TZ to those in UG by calculating *z*-scores and their corresponding *p*-values.

To answer the second research question, we created indicators of risk of contamination by source type in a color-coded matrix Bain *et al*. (Supplementary Material Tables S3 and S4) [13]. Importantly, the studies included in this meta-analysis included measurements from both the wet and dry seasons. For the specific criteria used for inclusion in the meta-analysis, see Bain *et al*. [13]. To do so, we calculated the proportion of samples detecting ≥ 10 *E. coli* per 100 mL for each study included in the meta-analysis (*n* = 319). This specific meta-analysis was used as it included data for surface water sources and did not restrict to studies that assessed quality of stored water, as done in subsequent studies [14,16]. We then calculated descriptive statistics for these proportions including range, median, mean and standard deviation for each water source type (unprotected springs, unprotected dug wells, rainwater, and borehole), and assigned each source type a general risk level from low to very high. 

### Ethical Review

These two projects underwent ethical review separately. The Ugandan study was approved by the University of Washington (HSD # 07-5209-J01), Mbarara University of Science and Technology and by the Uganda National Council for Science and Technology. The Tanzania study was approved by Michigan State University (IRB# x15-423e) and the Tanzanian COSTECH (#2015-207-NA-2015-149).

## 3. Results

Table 1 shows that a significantly higher proportion of households in TZ (71%) use the same primary water source type year round compared to UG (50%; *z* = 2.86, *p* < 0.01). Among households that switched source type from wet to dry season, no households in TZ switched to a higher risk source type, while 20% of households in UG switched to a higher risk source type. Also in TZ, 9% of households switched to a lower risk primary water source type, compared to a significantly higher 26% of households in UG (*z* = 3.04, *p* < 0.01).

In Table 2, unprotected dug wells (shown in red) had the highest proportion of samples with ≥10 *E. coli*/100 mL, followed by unprotected springs (orange) and then rainwater (yellow). Boreholes (green) had the lowest average proportion of samples exceeding the threshold of ≥10 *E. coli*/100 mL.

**Table 1:** Households using the same or a different source type from wet to dry season.

	Switched to Higher Risk ± Source Type, *n* (%) ^§^	Switched to Lower Risk ± Source Type, *n* (%) ^§^	Used Same Source Type Year Round, *n* (%)
Tanzania	0 (0)	7 (9)	65 (71)
Uganda	17 (20)	22 (26)	46 (50)

^±^ For risk categories, see Table 2; ^§^ Households using source type “other” excluded.

**Table 2:** Descriptive statistics calculated in this study for samples with ≥10 *E. coli*/100 mL the using Bain *et al*. [13] (Supplementary Material Tables S3 and S4), and the resulting general risk level indicators.

Source Type	Bain *et al.* [13] *n* (studies)	Bain *et al.* [13] *n* (samples)	Mean (%)	Range (%–%)	Median (%)	SD (%)	Risk Level
Borehole	25	3651	32	0–90	32	26	Low
Rainwater	11	982	58	14–100	57	24	Medium
Unprotected springs	4	84	88	77–100	88	10	High
Unprotected dug well	14	867	94	38–100	97	20	Very high

Water source types are color coded to reflect their general risk level (green = low, yellow = medium, orange = high, red = very high).

During the wet season, only 33% (UG, *n* = 30) and 37% (TZ, *n* = 34) of households use a borehole as their primary source (Table 3). During the dry season, 50% (UG, *n* = 46) and 48% (TZ, *n* = 44) of households use a borehole as their primary source. In evaluating changes in source type, across both samples, 34% of households changed source type seasonally. From wet to dry season, 24% (UG) and 8% (TZ) of households switched from a source with higher risk of contamination to a source with lower risk of contamination (shown in hues of blue no such highlighting in Table 3). For example, 16 households (UG) switched from rainwater to a borehole, and five households (TZ) switched from unprotected dug wells to a borehole. In comparison, only 12% (UG) and 0% (TZ) of households switched from a source with lower risk of contamination to a source with higher risk of contamination (shown in hues of pink). Only six households out of all 184 shifted from a groundwater source in the wet season to a surface water source in the dry season.

**Table 3:** Primary household water source type from wet to dry season and general level of risk of fecal contamination.

**Uganda (*n* = 92)**							
	**Dry Season Water**						
		Borehole (B)	Rainwater (R)	Unprotected springs (US)	Unprotected dug well (UD)	Other (O)	Wet Season Sum
**Wet Season Water**	Borehole (B)	24	0	0	6	0	30
	Rainwater (R)	16	0	0	11	2	29
	Unprotected springs (US)	0	0	0	0	0	0
	Unprotected dug well (UD)	4	0	2	22	3	31
	Other (O)	2	0	0	0	0	2
	Dry Season Sum	46	0	2	39	5	92
**Tanzania (*n* = 92)**							
	**Dry Season Water**						
		Borehole (B)	Rainwater (R)	Unprotected springs (US)	Unprotected dug well (UD)	Other (O)	Wet Season Sum
**Wet Season Water**	Borehole (B)	34	0	0	0	0	34
	Rainwater (R)	0	0	0	0	0	0
	Unprotected springs (US)	2	0	16	0	1	19
	Unprotected dug well (UD)	5	0	0	25	4	34
	Other (O)	3	0	0	1	1	5
	Dry Season Sum	44	0	16	26	6	92
**Higher Risk of Contamination—Wet to Dry Seasons ^†^**			**Consistent Risk of Contamination—Wet to Dry Season ^†^**		**Lower Risk Contamination—Wet to Dry Seasons ^†^**		
		From B to other sources		Very high	From other sources to B		
		From R to other sources		High				
		Unknown		Medium	From UD to US		
				Low	Unknown		

^†^ Estimated from Bain *et al.* [13] data.

## 4. Discussion and Conclusions

Fewer households used lower risk boreholes in the wet season, compared to the dry season in this study. Interestingly, other research suggests that improved sources, including boreholes, have significantly lower levels of fecal contamination during the dry season [15]—Which is when more households used these sources. Twenty six percent of households in Uganda and nine percent in Tanzania switched to a lower risk primary water source type in the dry season. The importance of these findings are two-fold. First, these findings suggest that one pathway through which water-related disease prevalence may be higher in the wet season is the change in source type across seasons. Second, these findings suggest that even when households have access to likely less contaminated sources (e.g., a borehole), they tend to choose to use other sources, including surface water, when they are available during the wet season.

This study has limitations. First, we measured primary water sources used across seasons. In reality, households may use more than one water source and use separate sources for different household purposes. Second, all data on water source use were self-reported and thus prone to relevant sources of bias. In addition, we did not strictly survey the household member in charge of obtaining water for the household. This may have led to some misclassification in our data. However, it is unlikely that this misclassification was differential by season or setting. Future studies, however, would perhaps have more reliable data if they strictly survey household water managers. We also relied on water quality findings included in a meta-analysis to calculate the average proportion of samples exceeding a water quality threshold and to create risk categories for source types. We did not further exclude studies based on study quality or season when sampled. For some water source types, numerous studies were included, while for other types, fewer studies were included. Full descriptive statistics were provided to give the reader an idea of the distribution of the values. Additionally, the raw data from the meta-analysis used here to create the general risk indicators did not differentiate between wet and dry seasons. In the future, water quality measurements should optimally be collected alongside source type usage across seasons. Last, in Tanzania, the study was conducted in a community that received a new borehole funded by the Tanzania Partnership Program, and thus respondents may have been more likely to report using water from that source, in an effort to appear grateful.

It is clear that households’ preferred water source often varies across seasons, with clear implications for health. Future research could qualitatively explore the decision-making process and drivers of those decisions for households, including the household members involved in such decisions and whether distance to source plays a role (e.g., nearby ponds may form in the wet season which evaporate, leaving only a distant borehole). Since the study communities here consist primarily of agro-pastoralists, it would also be useful to understand whether access to water for livestock is a major contributor to shifts in water sources. In addition to understanding the reasons for changing sources, future research could show changes in water source at a finer temporal scale. For example, how frequently do households obtain water from different sources within a week or a month? Are different sources used for different household purposes? Understanding the frequency and drivers for changes in water sources may be useful in planning efforts for water provision and the complex decision-making processes that households may engage over time. Such future findings may also shed light on the dynamic burden of fetching water and the tenuous nature of access, even in communities with ‘access to improved water’. Future studies could also measure contamination of sources used across time and assess water storage, which may be significantly higher than source levels [16].
